# Advertising support in healthcare settings for survivors of sexual violence: findings from a population-based survey in England

**DOI:** 10.3389/frph.2025.1642585

**Published:** 2025-07-31

**Authors:** Aliyu Abubakar, Jonathan D. C. Ross, Rachel J. Caswell

**Affiliations:** Department of Sexual Health and HIV Medicine, University Hospitals Birmingham, NHS Foundation Trust, Birmingham, United Kingdom

**Keywords:** sexual violence, support, sexual and reproductive health, advertisement, awareness

## Abstract

**Introduction:**

Sexual violence (SV) is common, but accessing appropriate support is frequently a challenge. Understanding how best to advertise healthcare support after SV could potentially improve access and long-term outcomes. This study investigates factors associated with increasing the visibility of advertisements for SV support in healthcare settings, and variations in the relevance of advertisements to different population sub-groups.

**Methods:**

An online, population-based survey was conducted in England. The survey collected demographic data, history of whether they had seen SV support information and its perceived relevance.

**Results:**

Overall, 2,007 respondents aged 18 years and above completed the survey. On multivariate analysis, younger age groups were significantly more likely to report seeing SV support advertisements compared to those aged 55 or older—individuals aged 18–24 years (AOR = 2.23, 95% CI = 1.36–3.65) and 25–34 years (AOR = 2.26, 95% CI = 1.43–3.57). Ethnicity and prior experience of SV were also significant predictors, with White respondents more likely to have seen the advertisements (AOR = 5.52, 95% CI = 1.81–16.85), as were those with a history of SV (AOR = 1.66, 95% CI = 1.34–2.05). Respondents aged 18–24 years (AOR = 3.29, 95% CI = 1.80–6.04) and 25–34 years (AOR = 2.32, 95% CI = 1.34–4.04) reported SV support information to be more relevant to them than those aged 55 or older, as did individuals with a history of SV (AOR = 1.82, 95% CI = 1.42–2.33).

**Conclusions:**

The findings suggest younger people and those of White ethnicity and with a history of SV are more likely to see SV support information and perceive it as relevant. Advertising strategies targeting high-risk populations are essential to ensure equitable access to care.

## Introduction

Sexual violence (SV) is a significant public health and societal issue with profound physical, psychological, and socioeconomic consequences, which include unintended pregnancy, genital/physical injuries, chronic pelvic pain, and increased risk of sexually transmitted diseases (STDs), including HIV (1). Sexual and reproductive health services (SRHS) are key settings that provide safe and supportive care following SV, including the provision of emergency contraception, prophylaxis against hepatitis B and HIV, testing for HIV and other STDs, and offer a pathway into other support services (2).

Despite the widespread nature of SV, survivors often face barriers to accessing timely healthcare; for example, only 21% of women in the United States reported seeking immediate medical care following SV (3). In the United Kingdom, approximately 85% of SV incidents are not formally disclosed to the appropriate authorities (4), and a National Health Service survey in England found that 72% of respondents were unaware of Sexual Assault Referral Centres (SARCs), which offer forensic and medical care after SV (5). Raising awareness of available services through public-facing materials may help address these gaps and support survivors to access the care they need.

Advertising campaigns can raise awareness of specific health issues and improve uptake of services (6). Health communication campaigns have previously demonstrated success in increasing service uptake and reducing stigma in other areas of health (7–9). For example, domestic abuse support advertising campaigns in emergency departments and maternity services in five hospitals in England have improved recognition of abuse and increased disclosures (9), while mental health campaigns targeting stigma and promoting help-seeking have successfully shifted public attitudes towards seeking care (10, 11).

Previous research on sexual violence support advertising suggests that increasing the visibility of the advertisements through optimal placement and using inclusive wording and imagery that addresses rape myths can help potential service users feel more confident in identifying as candidates for care (12–16). However, the visibility and perceived relevance of SV support advertising in healthcare settings remain underexplored.

The effectiveness of health advertising is influenced by message framing and the medium of dissemination (17). Tailored language and representation of diversity can significantly affect whether viewers perceive the information as applicable to them (18). In the context of SV, socio-cultural barriers, including stigma, fear of judgment and societal stereotypes, increase the challenges to effective advertising (19)*.* To address these barriers, public-facing materials need to be carefully worded to help individuals recognize themselves as appropriate candidates for care (20). However, few studies have explored the effectiveness of advertising for SV support, and strategies that most effectively reach and engage different population groups remain seldom evaluated^1^. Awareness and engagement with health-related information can vary based on background and personal experiences; for example, younger individuals may be more engaged with available health information, possibly due to increased use of technology to access it (21). There are also concerns that certain groups, such as individuals from minority ethnic backgrounds, may have less exposure and awareness of relevant information [(21), (see text footnote 1)].

This study uses data from a population-based survey conducted in England to investigate which factors are associated with the visibility of advertisements for SV support in healthcare settings and whether advertisements are perceived as relevant by different population groups. Such findings can be used to inform the design of future advertising campaigns directed at those who have experienced SV.

## Methods

### Study design

An online, population-based survey was conducted in England from 19 to 31 January 2022. The survey, complying with the Consensus-Based Checklist for Reporting of Survey Studies (22), was distributed to a panel of approximately 600,000 English residents hosted by a professional marketing and data company (Dynata Global UK Ltd). Detailed study methodology and initial findings have previously been published (23).

A pilot was conducted from 6 to 12 January 2022 with 300 participants, which assessed the questionnaire's consistency, completion rate, validity, and reliability, leading to minor revisions and pilot data included in the final analysis.

### Sampling strategy and recruitment

The survey was open to individuals aged 18 years and older, using open recruitment with quota sampling targets until a minimum of 2,000 participants was reached. Age quotas included a minimum of 5% participants aged 18–24 years and a maximum of 10% aged 55 or older to ensure representation. For ethnicity, the target was a maximum of 85% White British, with at least 1% self-identified Black participants, reflecting England's national census data and ensuring representation of minority groups (24).

### Data collection

The survey collected data on participants’ demographic characteristics and history of SV. Findings related to knowledge of SRHS and preference for which services to attend following SV have previously been published (23). This study focuses on questions related to advertising of SV support, specifically:

• *When you have been in healthcare settings, do you think you have seen information on display (e.g., on posters, leaflets, screens) about where to get help after sexual violence?*
• *Was the information (e.g., on posters, leaflets, screens) relevant to you and your background? (see* Supplementary File 1 *for detailed survey questions).*

### Data analysis

Descriptive statistics were used to summarize the participants' characteristics, and Chi-square tests were conducted to examine differences in the visibility and perceived relevance of advertising strategies across sociodemographic and population groups. Variables with *p* < 0.20 at bivariate analysis were included in the multivariate logistic regression to investigate factors associated with increased visibility and relevance of advertisements for SV support services among different population groups. A *p*-value of <0.05 was considered statistically significant in the final multivariate model. Analyses were conducted using SPSS 28.0 (SPSS Inc.®, Chicago, Illinois, USA).

### Ethical considerations

Ethical approval was obtained (Health Research Authority approval granted on 27 July 2021, substantial amendment no. RG_19-102 SA01). Informed consent was obtained from respondents online prior to accessing the survey. The initial survey page outlined the nature and purpose of the survey and contact information to access support after SV was provided. The data were anonymized before being sent to the research team.

## Results

### Participants characteristics

A total of 2,007 participants completed the survey. The majority self-identified as White (85.5%), female (62.3%), and heterosexual (87.0%). Additionally, 14.2% reported a disability, 72.4% were in an intimate relationship, and 27.1% reported a past experience of SV (see Supplementary File 2).

### Visibility of advertisements for support following SV

Overall, 51.8% of respondents reported having seen advertisements for SV support services in healthcare settings. Of the 1,960 participants who provided information on SV history, 62.8% of those with a history of SV reported seeing advertisements, compared to 46.8% without such history (Table 1).

**Table 1:** Overall visibility of advertisements for support following SV in a healthcare setting.

Seen SV support advertisement	“No’ History of SV *n* = 1,339 (%)	History of SV *n* = 621 (%)	Total *n* = 1,960 (%)
Yes	626 (46.8)	390 (62.8)	1,016 (51.8)
No	713 (53.2)	231 (37.2)	944 (48.2)

SV, sexual violence. Denominator is *n* = 1,960, as the *n* = 47 who did not respond to the question about their previous history of SV were excluded.

Respondents aged 18–24 years and 25–34 years were more likely to report seeing SV support services advertisements in healthcare settings than those aged 55 years or older, with adjusted odds ratios (AOR) = 2.23 (95% CI = 1.36–3.65) and AOR = 2.26 (95% CI = 1.43–3.57), respectively. Ethnicity, being in an intimate relationship and prior experience of SV were also significant predictors—White ethnicity AOR = 5.52 (95% CI = 1.81–16.85); respondents in intimate relationships AOR = 1.65 (95% CI = 1.34–2.05); history of SV AOR = 1.66 (95% CI = 1.34–2.05) (Table 2).

**Table 2:** Predictors of visibility and relevance of sexual violence support advertisements in healthcare settings Among respondents.

Variables	Visibility of advertising strategies					Relevant information from advertising strategies				
	Crude OR	Adjusted OR	95% Confidence Interval		*p*-value	Crude OR	Adjusted OR	95% Confidence Interval		*p*-value
			Lower	Upper				Lower	Upper	
Age (Years)										
18–24	2.22	2.23	1.36	3.65	***p*** **=** **0.001**	3.81	3.29	1.80	6.04	***p*** **<** **0.001**
25–34	2.10	2.26	1.43	3.57	***p*** **<** **0.001**	2.50	2.32	1.34	4.04	***p*** **=** **0.003**
35–44	1.53	1.71	1.10	2.65	***p*** **=** **0.018**	1.40	1.39	0.82	2.36	*p* = 0.225
45–54	0.87	0.99	0.63	1.54	*p* = 0.959	0.96	0.96	0.56	1.64	*p* = 0.879
≥55 (RC)										
Ethnicity										
White	5.37	5.52	1.81	16.85	***p*** **=** **0.003**					
Asian	0.78	0.71	0.37	1.35	*p* = 0.291					
Mixed	0.48	0.63	0.35	1.14	*p* = 0.124					
Black	1.16	1.20	0.85	1.70	*p* = 0.300					
Others (RC)										
Intimate relationship										
Yes	1.71	1.65	1.34	2.05	***p*** **<** **0.001**	1.60	1.43	1.10	1.85	***p*** **=** **0.007**
No (RC)										
Disabled										
Yes	1.57	1.37	1.04	1.82	***p*** **=** **0.027**					
No (RC)										
Previous SV										
Yes	1.92	1.66	1.34	2.05	***p*** **<** **0.001**	2.17	1.82	1.42	2.33	***p*** **<** **0.001**
No (RC)										

OR, odds ratio; RC, reference category; SV, sexual violence.

Bold *p*-value showed statistical significance.

### Relevance of advertisements for support following Sv

Younger respondents were more likely to find SV support services advertisements relevant—for those aged 18–24 years, AOR = 3.29 (95% CI = 1.80–6.04) and 25–34 AOR = 2.32 (95% CI = 1.34–4.04). Similarly, respondents in intimate relationships were more likely to report the advertising information as relevant compared to those not in a relationship (AOR = 1.43, 95% CI = 1.10–1.85), as did individuals with a history of SV (AOR = 1.82 (95% CI = 1.42–2.33) (Table 2). Significant differences in the visibility and perceived relevance of SV support services advertisements were found across different population groups (see Supplementary File 3).

## Discussion

We identified several factors associated with the visibility and relevance of advertisements for support following SV. Notably, only around half of respondents in this study reported seeing SV support service advertisements in healthcare settings, indicating limited use of advertisements and/or their visibility. This aligns with findings from the National Health Service survey in England that showed 72% of the public are unaware of Sexual Assault Referral Centres (5), reflecting broader challenges in public awareness of available SV support services.

Significant differences were identified in the visibility and perceived relevance of advertising resources between different population groups, highlighting a need for more targeted and inclusive advertising strategies to address disparities and ensure equitable access to care. Existing health inequalities with regard to access to care affect some groups more than others, and advertising strategies should ensure they are reaching intended audiences (25).

Ethnic differences were evident, with White participants being significantly more likely to report seeing SV support information than individuals from other ethnic backgrounds. This is consistent with previous research indicating that minority ethnic groups often face barriers in accessing health information, possibly because of language difficulties, cultural stigma, and/or stereotyping (26). These disparities raise potential concerns about the appropriateness of SV support advertisements for minoritized groups and highlight a need to ensure advertisements are culturally sensitive and inclusive.

Younger individuals were also more likely to report seeing SV support advertisements and perceive them as relevant than older age groups. This aligns with findings from previous studies, in which younger populations tend to engage more with sexual health-related information and services (21). It may also reflect differences in sexual health engagement patterns and digital literacy among younger age groups. Current advertising strategies may be more effective in reaching younger populations, potentially due to differences in placement, design, or accessibility of these resources.

Individuals in intimate relationships were more likely to see and find advertisements relevant, possibly due to a heightened awareness of relationship dynamics that prompt engagement with services. Similarly, the perceived relevance of the advertisements among those with a history of SV may reflect greater awareness and underscore the importance of advertising in engaging with SV survivors. Prior research has shown that survivors of SV are more likely to seek and engage with support services when available information is perceived as being confidential and relevant to their background (20).

The study included a relatively large sample size, with participant demographics broadly comparable to national census data for ethnicity, disability, and sexual orientation, with the use of quota sampling (27). However, the sample may be biased toward individuals with access to and proficiency in using technology, potentially overrepresenting higher than average socioeconomic groups (28). Additionally, the survey focused on advertising in healthcare settings, excluding community and online advertising. It was also not possible to assess how effective advertisements were in facilitating attendance at services. Given the self-reported nature of the data, there is a possibility of recall bias, particularly in participants' ability to accurately remember exposure to advertising materials in healthcare settings. However, piloting of the survey instrument aimed to minimize this by ensuring that questions were clearly worded, consistently understood, and capable of capturing relevant past experiences (29).

The study findings emphasize the need for a more inclusive approach to designing and disseminating SV support services advertisements. Adopting a service user-informed co-design approach and tailoring advertisements to ensure greater visibility and relevance among underrepresented populations is likely to enhance their effectiveness. This study did not assess the effect of specific design features, content, or formats of advertisements and how this impacts visibility and acceptance, which limits the ability to determine which characteristics drive visibility and relevance across population groups. Prior research in healthcare and SV advertising has shown that design elements such as message framing, visual appeal, tone, and the use of inclusive imagery can significantly influence how audiences interpret and engage with health messaging (12–18).

Future research should build on this by examining how specific design and content features of advertisements influence engagement, particularly among underrepresented populations. This includes evaluating how elements such as tone, message framing, imagery, and delivery channels affect comprehension, relevance, and acceptance. Such work would provide comprehensive insight into not just who sees and values advertisements, but also why certain advertisements resonate with individuals. Exploring these dimensions will be essential for developing advertising campaigns that are both equitable and impactful in reaching those affected by SV.

## Data Availability

The raw data supporting the conclusions of this article will be made available by the authors, without undue reservation.

## Footnotes

^1^Abubakar A, Ayinde O, Ross JDC, Jackson L, Caswell RJ. Evaluating advertising strategies for sexual and reproductive health care after sexual violence: a systematic review. Trauma, Violence, & Abuse.
