# Supplementary material for: Advertising support in healthcare settings for survivors of sexual violence: findings from a population-based survey in England
Source: Front Reprod Health. 2025 Jul 31;7:1642585. doi: 10.3389/frph.2025.1642585 (PMC12350387; doi:10.3389/frph.2025.1642585)
Supplement: Supplementary file 1 [file Table1.docx]

Supplementary file 1: Survey Questions

This survey was conducted online and based on the text below. Minor changes were made after piloting. Questions related to knowledge of SRHS and preference for which services to attend following SV have previously been published <https://doi.org/10.1136/bmjopen-2023-073204> . This study focuses on questions related to advertising of SV support in healthcare settings.

We are collecting this information as part of a research project to improve the way NHS sexual health clinics respond to people who have been subjected to sexual activity that they did not agree to (another name for this is sexual violence which includes rape, sexual assault and sexual abuse).

You can complete this survey if you

- Are aged 18 years or above
- Live in England

This survey does not ask for detailed information on previous sexual experiences; it is looking for your ideas on how we can improve the way we run services.

Although many people have experienced sexual violence and abuse in their lifetime, you do not need to have experienced sexual violence in order to complete this survey.

The survey is anonymous; we will not know who you are. Only a small team at University of Birmingham will have access to this information and even then, they will not be able to identify you. Your responses will be kept private and secure. We want sexual health services to be accessible to all and your answers will help us understand how to do this better.

Data from the research will be combined in final reports and anonymised results from this survey may be used in publicly accessible reports and articles. Please be aware that it will not be possible to retract your survey contribution once you have pressed the ‘Finish’ button. At the end of the research project your survey response will be destroyed.

If you have any questions about the survey, the research more broadly or want to find out more, please contact [removed for peer review]

Consent

Please read the consent statement below.

I have read the information about this research and understand what it is about.

I understand that I have the right to refuse to answer particular questions and that I can end

IRAS 266583 20.11.21 V3 1

the survey at any time.

It is clear to me that I cannot ask for my survey response to be removed once I have clicked the 'Finish' button.

I agree to take part in this research project.

I consent to participate in this survey.  *Required* *Yes*

*No*

The survey should take no more than 15 minutes but you can stop at any time if you want. All of the responsibility for sexual violence lies with the perpetrator(s), anyone can be affected and there is support available. If you have been affected by any of these issues, please know there is support for you:

[https://www.nhs.uk/live-well/sexual-health/help-after-rape-and-sexual-](https://www.nhs.uk/live-well/sexual-health/help-after-rape-and-sexual-assault/?tabname=advice-and-support) [assault/?tabname=advice-and-support](https://www.nhs.uk/live-well/sexual-health/help-after-rape-and-sexual-assault/?tabname=advice-and-support)

[https://www.samaritans.org](https://www.samaritans.org/)

[https://rapecrisis.org.uk](https://rapecrisis.org.uk/)

[https://rapecrisisni.org.uk](https://rapecrisisni.org.uk/)

[https://www.thesurvivorstrust.org](https://www.thesurvivorstrust.org/)

[https://malesurvivor.co.uk](https://malesurvivor.co.uk/)

[https://www.nationaldahelpline.org.uk](https://www.nationaldahelpline.org.uk/)

There are three sections to this survey

1. Confidential background information about you for us to understand the range of people completing the survey
2. Personal information that includes asking whether you have ever been subjected to sexual violence or sexual abuse (The questions in this section have been carefully designed so as to be as sensitive as possible. As with any part of this survey, it is your choice whether you answer these questions)
3. What you think is, or might be, important for those attending a sexual health clinic after sexual violence or sexual abuse.

IRAS 266583 20.11.21 V3 2

Section1

1. Which category below includes your age? 18-24

25-34

35-44

45-54

55 or older

1. What is your ethnicity? Choose one option that best describes your ethnic group or background.

White - British White - Irish

Any other white background Mixed white and black Caribbean Mixed white and black African Mixed white and Asian

Any other mixed background Asian or Asian British Indian Asian or Asian British Pakistani Asian or Asian British Bangladeshi Any other Asian background Black or Black British Caribbean Black or Black British African

Any other Black background Chinese

Arab

IRAS 266583 20.11.21 V3 3

Any other ethnic group not stated

1. How would you describe your gender? Female Male Non-binary Prefer not to say I

describe my gender in another way (please specify) …….

1. Sex assigned at birth: male female intersex Prefer not to say
2. Sexual Identity/Sexual Orientation (select all that apply): Bisexual

Gay/lesbian Heterosexual/straight Pansexual

Queer

Don’t know

Prefer not to say

Something else. Please say what

1. Do you consider yourself a disabled person? Yes No
2. Are you currently in an intimate relationship? Yes No Not sure Would rather not say
3. What is your legal marital or registered civil partnership status? Never married and never registered a civil partnership

Married

In a registered civil partnership Separated but still legally married

Separated but still legally in a civil partnership Divorced

Formerly in a civil partnership which is now legally dissolved Widowed or surviving partner from a registered civil partnership

IRAS 266583 20.11.21 V3 4

1. Which part of England do you live in? North East

North West Midlands

East of England London

South East South West

I do not want to say

Section 2

This section asks for personal information including whether you have ever been subjected to sexual activity that you did not want or agree to like sexual violence or abuse (you can skip this section if you are concerned it may upset you, however we hope the questions are asked in a safe way). The reason for asking is to understand whether people who have personally experienced this trauma answer differently to those who haven’t. The questions are not looking for details of the sexual violence or abuse.

This is confidential and anonymous. If you would like support please contact [link] (to above website list of services)

1. Have you heard of Sexual health clinics (also known as GUM/genitourinary clinics/Sexual and Reproductive Health clinics)?

Yes No Not sure

If yes or not sure:

1. Have you attended a sexual health clinic? Yes, within the last 5 years

Yes, but more than 5 years ago No

Not sure

IRAS 266583 20.11.21 V3 5

I would prefer not to say

1. Are you aware that Sexual health clinics offer support after sexual violence or abuse? Yes No Not sure
2. In your opinion, where would you or someone like you go to get support after sexual violence or abuse? Choose up to 3, with 1 being most likely place to go. (We understand you may want to choose more than 3 but it is helpful for us to understand what your top choice(s) would be)

Rape Crisis or other similar charity GP/Family doctor

Sexual Assault Referral Centre or SARC/Haven

Sexual Health clinic (Sexual and Reproductive health clinic/GUM) Emergency department (also known as A&E)

Police (e.g., by calling 999, 111 or attending police station) To a friend/family member

Choose not to go anywhere or tell anyone Somewhere else. Please state

1. Have you ever been subjected to sexual activity that you did not want or agree to like sexual violence or abuse?

Yes No Not sure Prefer not to say

If no, or prefer not to say, end section and go to Section 3 If not sure add question

1. I am not sure because (select best fit):

The words I would use to describe what happened are not sexual violence or abuse* (if yes, add definition of consent*, and carry on with the section)

IRAS 266583 20.11.21 V3 6

I had initially agreed to the sexual activity then changed my mind (if yes, add definition of consent*, and carry on with the section)

I cannot remember what happened (this could be for a variety of reasons e.g., because I was too young, under the influence of alcohol/drugs or I find it difficult to think about it) (if yes add definition of consent* and carry on with the section)

1. Some other reason (– if ticked – Do you wish to carry on with this Section? Y N )

*Sexual violence is any sexual activity that takes place without someone’s consent. If someone consents to sex, it means they agree by choice, and also that they have the freedom and capacity to make that choice. People also have the right to change their minds at any time.

1. Did this unwanted sexual activity happen once more than once
2. I was sexually abused as a child Yes No Not sure Prefer not to say 23 After it happened, the first time I told a nurse or a doctor about it was within:

One week

After one week and within the first 6 months After 6 months and within the first year Between one year and 10 years

More than 10 years

Never (I have never told a nurse or doctor about this)

(If has told a nurse or doctor i.e. has responded to the above with any option apart from

‘Never’)

24 Please answer yes, no or not sure to the following questions. Thinking about the most recent time (or the only time if that applies):

I have told my GP (family doctor)

I have told a nurse or doctor at a SARC (sexual assault referral centre, also known as the Havens in London)

IRAS 266583 20.11.21 V3 7

After the unwanted sexual activity I told a nurse or doctor at the Emergency department (also known as A+E)

Section 3

When patients attend sexual health clinics, they are asked a range of health-related questions. We want to understand your views on being asked about sexual activity that was not agreed to.

Please assume that the following would *always* apply:

- A question asking about sexual violence or abuse would only be asked when safe to do so, for example when no one else was present.
- Before asking you would be advised that you do not have to answer the question if you don’t want to
- Before asking, you would be advised what would happen if you answer ‘yes’, including how your information is kept confidential unless vulnerable people are at serious risk of harm
- Any nurse/doctor asking would have had training in how to respond to a disclosure and how to support someone safely

28 If these things were in place, what is your view on whether patients attending a Sexual Health clinic are asked about sexual violence or abuse?

Should be asked on each visit Should be asked less frequently

Should never be asked (should be left with patient to decide whether they want to bring it up or not)

28 If answers ‘Should never be asked (should be left with patient to decide whether they

want to bring it up or not)’ add… Because (indicate top reason): It is not something relevant to ask in a Sexual Health Clinic

It will take up consultation time and distract from the health issue they had attended for The question may be upsetting for the patient

IRAS 266583 20.11.21 V3 8

It should be left with the patient to decide whether they want to bring it up or not Other

28 If answered ‘Should be asked less frequently’, please now identify how often: Should be asked on the initial visit only

Should be asked more than just the initial visit but at healthcare professional’s discretion e.g., if attending multiple follow-up visits should not be asked on each occasion but if a gap in attendance should be re-asked

Should only be asked by nurse/doctor when they suspect something might have happened e.g., injuries, chronic abdominal pain, distress

29 In what way would you prefer to be asked about sexual activity that was not agreed to like sexual violence and abuse?

It is preferable to be asked face to face with a doctor or nurse

It is preferable to be asked while filling in a sexual health questionnaire (e.g., electronically or on paper form) before seeing the doctor or nurse who would review my responses with me

I would like to be asked in both or either way – face to face and/or on a form I would prefer never to be asked

Other

During the COVID-19 pandemic more healthcare consultations have been carried out by telephone. Please assume that the following would *always* apply:

- A question asking about sexual violence or abuse would only be asked when safe to do so, for example when no one else was present (note this can be difficult on the phone but if there are any concerns the healthcare professional will not ask)
- Before asking you would be advised that you do not have to answer the

question if you don’t want to

IRAS 266583 20.11.21 V3 9

- Before asking, you would be advised what would happen if you answer ‘yes’ including how your information is kept confidential unless vulnerable people are at serious risk of harm
- Any nurse/doctor asking would have had training in how to respond to a disclosure and how to support someone safely

30 Do you think people should be asked during a telephone consultation if they would like support after sexual violence or abuse?

Yes (if the above points are ensured) No

Not sure

31 If answers no or not sure add… Because (choose top reason): It is not something relevant to ask in a Sexual Health Clinic

It will take up consultation time and distract from the health issue they had attended for The question may be upsetting for the patient

It should be left with the patient to decide whether they want to bring it up or not

It may not be safe for the person to be asked on the phone (even with the safety checks above)

It should only be asked face to face Other

32 Here are some wording examples that could be used by a doctor or nurse in asking everyone who attends a sexual health clinic about sexual violence and abuse.

Tick which you like best: (only allow one to be ticked ALONG with free text)

Many people are subjected to sexual abuse and sexual violence and therefore we ask all patients about this. Would you like to hear about the support available?

IRAS 266583 20.11.21 V3 10

Many people are subjected to sexual activity to which they haven’t wanted or agreed to and therefore we ask all patients about this. Would you like to hear about the support available?

Sometimes people are not sure whether they agreed to or consented to sexual activity, or something has happened that was upsetting, and therefore we ask all patients about this. Would you like to hear about the support available?

None of these

Other please give an example:

People may not tell a nurse or doctor about sexual activity that they did not agree to when they attend a healthcare setting. They may choose to find support independently.

33. When you have been in healthcare settings, do you think you have seen information on display (e.g., on posters, leaflets, screens) about where to get help after sexual violence?

34. Was the information (e.g., on posters, leaflets, screens) relevant to you and your background?

Thank you for your time in completing this survey.

If you have been affected by any of these issues, please know there is support for you:

[https://www.nhs.uk/live-well/sexual-health/help-after-rape-and-sexual-](https://www.nhs.uk/live-well/sexual-health/help-after-rape-and-sexual-assault/?tabname=advice-and-support) [assault/?tabname=advice-and-support](https://www.nhs.uk/live-well/sexual-health/help-after-rape-and-sexual-assault/?tabname=advice-and-support)

[https://www.samaritans.org](https://www.samaritans.org/)

[https://rapecrisis.org.uk](https://rapecrisis.org.uk/)

[https://rapecrisisni.org.uk](https://rapecrisisni.org.uk/)

https://[www.thesurvivorstrust.org](http://www.thesurvivorstrust.org/) [https://malesurvivor.co.uk](https://malesurvivor.co.uk/)

IRAS 266583 20.11.21 V3 11

https://[www.nationaldahelpline.org.uk](http://www.nationaldahelpline.org.uk/)

IRAS 266583 20.11.21 V3 12
