# Supplementary material for: Advertising support in healthcare settings for survivors of sexual violence: findings from a population-based survey in England
Source: Front Reprod Health. 2025 Jul 31;7:1642585. doi: 10.3389/frph.2025.1642585 (PMC12350387; doi:10.3389/frph.2025.1642585)
Supplement: Supplementary file 2 [file Table2.docx]

Supplementary File 2 Participants Demographic Characteristics

|  | **Total (% of total respondents) ^a^** | **Previous sexual violence ^b^** |
| --- | --- | --- |
|  |  |  |
| Age (years) |  | **p<0.001** |
| *18-24* | 113 (5.6%) | 44/106 (41.5%) |
| *25-34* | 607 (30.2%) | 234/596 (39.3%) |
| *35-44* | 634 (31.6%) | 198/618 (32.0%) |
| *45-54* | 423 (21.1%) | 115/413 (27.8%) |
| *55 or older* | 230 (11.5%) | 30/227 (13.2%) |
| Ethnicity |  | p=0.053 |
| *White* | 1715 (85.5%) | 532/1678 (31.7%) |
| *Asian* | 160 (8.0%) | 38/157 (24.2%) |
| *Mixed* | 58 (2.9%) | 24/57 (42.1%) |
| *Black* | 50 (2.5%) | 19/45 (42.2%) |
| *Other ethnic group* | 24 (1.2%) | 8/23 (34.8%) |
| Gender |  | **p<0.001** |
| *Female* | 1250 (62.3%) | 452/1216 (37.2%) |
| *Male* | 742 (37.0%) | 162/732 (22.1%) |
| *Non-binary* | 8 (0.4%) | 4/8 (50.0%) |
| *I describe my gender in another way* | 3 (0.1%) | 2/3 (66.7%) |
| *I prefer not to say* | 4 (0.2%) | 1/1 (100.0%) |
| Sexual orientation |  | **p<0.001** |
| *Heterosexual/straight* | 1746 (87.0%) | 496/1712 (29.0%) |
| *Bisexual* | 116 (5.8%) | 68/115 (59.1%) |
| *Gay/lesbian* | 70 (3.5%) | 24/69 (34.8%) |
| *Pansexual* | 21 (1.0%) | 14/20 (70.0%) |
| *Queer* | 3 (0.1%) | 1/3 (33.3%) |
| *Asexual* | 1 (0.0%) | 1/1 (100.0%) |
| *Prefer not to say or not sure* | 50 (2.5%) | 17/40 (42.5%) |
| Disabled |  | **p<0.001** |
| *Yes* | 284 (14.2%) | 166/275 (60.4%) |
| *No* | 1723 (85.8%) | 455/1685 (27.0%) |
| In an intimate relationship currently? |  | p=0.092 |
| *Yes* | 1454 (72.4%) | 468/1424 (32.9%) |
| *No* | 494 (24.6%) | 132/481 (27.4%) |
| *Not sure* | 32 (1.6%) | 13/31 (41.9%) |
| *Prefer not to say* | 27 (1.3%) | 8/24 (33.3%) |

*Note.* ***^a^*** Denominator is the total number of respondents n=2007. **^b^** Denominator is n=1960, as the n=47 who did not respond to the question were excluded; responses of “not sure” were treated as “yes” for analysis; data are reported as the n (%) of respondents in each subgroup answering “yes”; p-values are from Chi-square tests and bold p-values are significant at p<0.05.
