# Supplementary material for: Advertising support in healthcare settings for survivors of sexual violence: findings from a population-based survey in England
Source: Front Reprod Health. 2025 Jul 31;7:1642585. doi: 10.3389/frph.2025.1642585 (PMC12350387; doi:10.3389/frph.2025.1642585)
Supplement: Supplementary file 3 [file Table3.docx]

Supplementary file 3 Chi-square Tables of Factors Associated with Visibility of Sexual Violence Support Advertisements in Healthcare Settings and its Relevance to Respondents Visibility of Advertising Information (Chi-square tests)

| **Variable** | **Visibility of Adverting Strategies** | | **Test statistics**  **p value** |
| --- | --- | --- | --- |
|  | **Yes**  **n (%)** | **No**  **n (%)** |  |
| **Age (Years) (n = 2007)** |  |  |  |
| 18-24 | 68 (60.2) | 45 (39.8) |  |
| 25-34 | 385 (63.4) | 222 (36.6) | χ^2^ = 65.856 |
| 35-44 | 315 (49.7) | 319 (50.3) | **p < 0.001** |
| 45-54 | 177 (41.8) | 246 (58.2) |  |
| 55 or older | 93 (40.4) | 137 (59.6) |  |
| **Ethnicity (n = 2007)** |  |  |  |
| White | 888 (51.8) | 827 (48.2) |  |
| Asian | 77 (48.1) | 83 (51.9) | χ^2^ = 20.338 |
| Mixed | 40 (69.0) | 18 (31.0) | **p < 0.001** |
| Black | 29 (58.0) | 21 (42.0) |  |
| Other ethnic groups | 4 (16.7) | 20 (83.3) |  |
| **Gender (n = 2007)** |  |  |  |
| Male | 392 (52.8) | 350 (47.2) | χ^2^ = 1.067 |
| Female | 637 (51.0) | 613 (49.0) | p = 0.586 |
| Other/non-binary/prefer not to say | 9 (60.0) | 6 (40.0) |  |
| **Sexual Orientation (n=1957)** |  |  |  |
| Heterosexual/Straight | 882 (50.5) | 864 (49.5) | χ^2^ = 1.386 |
| Bisexual/Pansexual | 95 (68.8) | 43 (31.2) | p = 0.500 |
| Gay/Lesbian | 44 (60.3) | 29 (39.7) |  |
| **Intimate relationship (n=1980)** |  |  |  |
| Yes | 807 (55.5) | 647 (44.5) | χ^2^ = 27.360 |
| No | 222 (42.2) | 304 (57.8) | **p < 0.001** |
| **Disabled (n=2007)** |  |  |  |
| Yes | 174 (61.3) | 110 (38.7) | χ^2^ = 12.079 |
| No | 864 (50.1) | 859 (49.9) | **p = 0.001** |
| **Previous SV (n=1960)** |  |  |  |
| Yes | 390 (62.8) | 231 (37.2) | χ^2^ = 43.777 |
| No | 626 (46.8) | 713 (53.2) | **p < 0.001** |
| **SV** = Sexual Violence, **χ^2^ =** Pearson’s Chi-square test, **Bold p-value** showed statistical significance | | | |

Relevance of Advertising Information (Chi-square tests)

| **Variable** | **Adverting Information is Relevant** | | **Test statistics**  **p value** |
| --- | --- | --- | --- |
|  | **Yes**  **n (%)** | **No**  **n (%)** |  |
| **Age (Years)** |  |  |  |
| 18-24 | 54 (64.3) | 30 (35.7) |  |
| 25-34 | 305 (65.3) | 162 (34.7) | χ^2^ = 77.564 |
| 35-44 | 262 (56.2) | 204 (43.8) | **p < 0.001** |
| 45-54 | 128 (41.8) | 178 (58.2) |  |
| 55 or older | 51 (32.1) | 108 (67.9) |  |
| **Ethnicity (n = 1482)** |  |  |  |
| White | 685 (53.7) | 590 (46.3) |  |
| Asian | 57 (54.8) | 47 (45.2) | χ^2^ = 3.240 |
| Mixed | 29 (61.7) | 18 (38.3) | p = 0.519 |
| Black | 24 (57.1) | 18 (42.9) |  |
| Other ethnic groups | 5 (35.7) | 9 (64.3) |  |
| **Gender (n = 1482)** |  |  |  |
| Male | 307 (55.6) | 245 (44.4) | χ^2^ = 1.196 |
| Female | 487 (53.1) | 430 (46.9) | p = 0.550 |
| Other/non-binary/prefer not to say | 6 (46.2) | 7 (53.8) |  |
| **Sexual Orientation (n=1957)** |  |  |  |
| Heterosexual/Straight | 684 (53.2) | 602 (46.8) | χ^2^ = 9.185 |
| Bisexual/Pansexual | 75 (68.2) | 35 (31.8) | **p = 0.010** |
| Gay/Lesbian | 27 (54.0) | 23 (35.0) |  |
| **Intimate relationship (n=1948)** |  |  |  |
| Yes | 629 (57.0) | 474 (43.0) | χ^2^ = 15.088 |
| No | 163 (45.3) | 197 (54.7) | **p < 0.001** |
| **Disabled (n = 1482)** |  |  |  |
| Yes | 136 (54.2) | 115 (45.8) | χ^2^ = 0.005 |
| No | 664 (53.9) | 567 (46.1) | p = 0.944 |
| **Previous SV (n=1960)** |  |  |  |
| Yes | 324 (66.9) | 160 (33.1) | χ^2^ = 44.905 |
| No | 461 (48.3) | 493 (51.7) | **p < 0.001** |
| **SV** = Sexual Violence, **χ^2^ =** Pearson’s Chi-square test, **Bold p-value** showed statistical significance | | | |
